# Exploiting the Advantages of Molecular Tools for the Monitoring of Fungal Indoor Air Contamination: First Detection of *Exophiala jeanselmei* in Indoor Air of Air-Conditioned Offices

**DOI:** 10.3390/microorganisms7120674

**Published:** 2019-12-10

**Authors:** Xavier Libert, Camille Chasseur, Ann Packeu, Fabrice Bureau, Nancy H. Roosens, Sigrid C. J. De Keersmaecker

**Affiliations:** 1 Transversal activities in Applied Genomics, Sciensano, J. Wytsmanstraat 14, 1050 Brussels, Belgium; 2 Cellular and Molecular Immunology, Groupe Interdisciplinaire de Génoprotéomique Appliquée (GIGA), Université de Liège (ULg), Avenue de l′Hôpital, 1 (B34), 4000 Sart-Tilman, Belgium; 3 Mycology and Aerobiology, Sciensano, J. Wytsmanstraat 14, 1050 Brussels, Belgium

**Keywords:** *Exophiala jeanselmei*, indoor air contamination, real-time PCR, NGS, detection, public health, identification, molecular methods

## Abstract

Today, indoor air pollution is considered a public health issue. Among the impacting pollutants, indoor airborne fungi are increasingly highlighted. Most of the monitoring protocols are culture-based, but these are unable to detect the uncultivable and/or dead fraction or species suppressed by fast-growing fungi, even though this fraction could impact health. Among the contaminants suspected to be part of this fraction, *Exophiala jeanselmei* is an interesting case study. Known to be pathogenic, this black yeast grows in humid environments such as air-conditioning systems, where it has been previously detected using classical culture-based methods. However, until now, this fungus was never detected in indoor air in contact with these air-conditioning systems. This study shows the first detection of *E. jeanselmei* in indoor air collected from offices in contact with contaminated air-conditioning reservoirs. While its presence in indoor air could not be demonstrated with culture-based methods, it was found by real-time PCR and massive parallel sequencing. The latter also allowed obtaining a broader view on the fungal diversity in the tested samples. Similar approaches were applied on water samples collected from the conditioning reservoirs to trace the source of contamination. The comparison of results obtained with both methods confirmed that the molecular tools could improve indoor air monitoring, especially of dead and/or uncultivable contaminants or when competition between species could occur.

## 1. Introduction

Today, as most of their time is spent inside homes, offices, and other workplaces, people are increasingly impacted by a low indoor air quality. Among the different indoor pollutants inventoried, fungi and molds appear to be important airborne contaminants in workplaces, houses, and buildings, and they are suspected to have an impact on health, especially on respiratory diseases such as allergy, asthma, asthma exacerbation, or rhinitis. In this way, indoor fungal contamination is more and more considered as a public health issue by the scientific community [1,2,3,4,5,6] and by preventive health care actors such as environmental agencies [7,8] and the World Health Organization [9]. These fungal contaminants can originate from different sources, i.e., they can enter from outside environments, via windows, aeration, and ventilation, such as is the case for *Alternaria alternata* and *Cladosporium* [10,11,12,13,14] species, or they can occur in water contained in reservoirs in air-conditioning systems. One such example is *Exophiala jeanselmei*, a black yeast known as a pathogenic species associated, amongst others, with cutaneous infections, subcutaneous cysts, and systemic and nosocomial infections [15,16,17,18]. This species, living in humid and oligotrophic environments, is observed in feed, sludge, and stagnant water [19]. Recently, *E. jeanselmei* has been detected for the first time in outdoor and at low levels in indoor air sampled in 2012–2015 at different locations in the areas of arctic settlement Tiksi (Russian Arctic) [20]. In indoor environments, *E. jeanselmei* is also detected in sludge piping, water systems (pipes, bathroom, reservoir, sauna, etc.) and water reservoirs of air-conditioning systems [17,19,21,22]. For some other species, it has been postulated that the mechanism of indoor air contamination is linked to these water reservoirs of air-conditioning units or pipings [12]. However, although *E. jeanselmei* can be detected in humid indoor environments (i.e., water samples taken from water reservoirs of air-conditioning systems), it has never been found before in indoor air in contact with these air-conditioning systems during the air-conditioning water monitoring campaign. It can be hypothesized that this is due to the disadvantages of the currently used monitoring methods. Indeed, routine protocols for indoor air fungal monitoring are still based on culture, colony counting, and microscopic visualization. Therefore, the results of these methods are dependent on the culture conditions, such as the selection of the medium, species competition (especially a problem of suppression by fast-growing fungi during long-term incubation periods), growth conditions, and differences in terms of incubation time [23,24]. Moreover, culture-based protocols are not able to detect the uncultivable and/or dead fraction (i.e., fragments of mycelia or cell walls, dead cells, etc.) which could be airborne and collected during the sampling [23,24]. However, this fraction of suppressed or uncultivable species could have an impact on health and should, therefore, also be monitored; otherwise, important elements are missing to establish the scientific link between fungal airborne contamination and health problems. This could especially be true for *E. jeanselmei*. The monitoring of this fungus in water samples is complicated, as it needs up to 21 days to grow in culture [25], while being highly demanding in terms of culture conditions, i.e., growth medium chosen, competition between species, temperature, and humidity. Especially, this last factor can impede the detection of *E. jeanselmei* in indoor air, as the fact that it might occur in a desiccated form will hamper its cultivation, and hence its detection, using the classical monitoring procedures.

The use of molecular methods, such as polymerase chain reaction (PCR), real-time PCR (RT-PCR), and sequencing, could offer a solution to investigate the indoor airborne fungal community, including this suppressed, uncultivable, and/or dead fraction [23,24,26,27,28,29,30]. We have previously developed a qRT-PCR-based assay for the detection of *E. jeanselmei* in water samples [31]. In the present study, we have tried this method on air samples collected with the Coriolis^®^ µ air sampler from different offices in contact with air-conditioning reservoirs contaminated with *E. jeanselmei*. The aim was to investigate the hypothesis that this species is occurring in indoor air as an out-competed or uncultivable and/or dead fraction, i.e., that it is not detectable with the classical monitoring methods based on culturing, but that it can be detected using molecular, i.e., DNA-based, technologies. To demonstrate that massive parallel sequencing could improve our insight into the fraction of uncultivable fungi, the RT-PCR analysis was complemented with massive parallel sequencing of the DNA extracted from the air and water samples.

## 2. Materials and Methods 

### 2.1. Sampling

All the samples (i.e., air and water samples) were collected between February 12th and March 13th, 2015 in Brussels, Belgium.

The reservoir of water from five air-conditioning systems, as seen in Table 1, for which previous monitoring results had pointed to *E. jeanselmei* contamination, was investigated using the sampling protocol of water from air-conditioning reservoirs previously described [32]. In short, 1 L of water situated between 1 cm and 5 cm above the bottom of the water reservoir situated in the pulse group (PG, i.e., the part of the air-conditioning system containing the machinery to pulse the air and the water-reservoir needed to maintain the required hygrometry level) was put into a sterile Duran bottle. All samples were kept at 4 °C until their analysis.

In addition to the water sampling, air sampling was done in the offices connected to the five air-conditioning systems of which water samples were collected (PG1 to PG5). For each of the air-conditioning systems, three offices were selected based on their distance to the PG, i.e., the closest (-1) and most distantly located (-3) office relative to the PG and one office in between the two other offices (-2). Each sample was taken in duplicate. The indoor air samples were collected with a Coriolis^®^ µ air sampler (Bertin Technology, Motigny-le-Bretonneux, France) following the sampling protocol reported by Libert and colleagues in 2015 [33].

### 2.2. Classical Analysis

The protocol used for the classical analysis (i.e., culture and fungal determination) of the water and air samples, collected with the Coriolis^®^ µ air sampler, has been previously described [31,32,33]. According to the protocols, the incubation time ranged between five and 21 days as recommended for the detection of major indoor air species and for the detection of *E. jeanselmei*. After five days of incubation, the plate was analyzed as it was recommended for the detection of indoor air species. After this microscopic analysis, the plate was reincubated for 16 days (total incubation time 21 days) as is recommended for the detection of *E. jeanselmei*.

### 2.3. DNA Extraction

The protocol used for the DNA extraction from water [31] and indoor air samples [33] was previously described. This extraction protocols have been optimized in order to avoid inhibition in downstream PCR-based analysis. Genomic DNA amount and purity were evaluated with a Nanodrop^®^ 2000 (Thermo Scientific, Wilmington, DE, USA).

### 2.4. RT-PCR Screening

The analyses of DNA extracted from air and water samples were performed in duplicate in two independent runs. The RT-PCR assay used was the SYBR^®^Green Ejeanselmei_ITS assay earlier reported for the detection of *E. jeanselmei* in water [31]. This assay uses the SYBR^®^green PCR Mastermix (Diagenode, Liège, Belgium). The sequences of the primers used (targeting the internal transcribed spacer region (ITS)) are provided by Libert and colleagues [31]. The primers were purchased from Eurogentec (Liège, Belgium). Each run was done on a CFX TouchTM Real-Time PCR detection System equipped with CFX Manager software V. 2 (Biorad, Temse, Belgium) and was carried out with 5 µL of a positive control (PC), corresponding to 200 theoretical copies of gDNA of *E. jeanselmei* strain BCCM/IHEM 4740 purchased from the BCCM/IHEM collection of Sciensano in Brussels (Belgium). A “no template control” (NTC), i.e., using Gibco^®^ DNase, RNase, Protease free pure water (Life Technologies, Gent, Belgium) as template, was also added in order to verify that no contamination occurred.

In order to confirm the identity of each RT-PCR amplicon, Sanger sequencing analysis was performed on an ABI3130xl Genetic Analyzer apparatus (Applied Biosystem, Life Technologies, Gent, Belgium) with the BigDye Terminator v3.1 cycle sequencing kit (Applied Biosystems) according to the manufacturer’s instructions. Each sequence was identified by comparison to the sequences available in the NCBI database using the BLASTn tool (http://blast.ncbi.nlm.nih.gov/).

### 2.5. Massive Parallel Sequencing

To confirm the identity of RT-PCR amplicons, but also to demonstrate that massive parallel sequencing could be useful for the investigation of uncultivable and dead species present in the air and water samples, all the samples were sequenced using an Illumina MiSeq instrument.

Because a massive parallel sequencing analysis using the Illumina MiSeq with a MiSeq reagent kit v3 reaches its optimal performance with fragments no longer than 600 bp and because the fungal ITS region has a length around 800 bp, the PCR amplicons for library preparation were prepared for both the ITS-1 and ITS-2 region using DNA extracted from different environmental (water and air) samples. Hereto, amplification with the universal forward (f) and reverse (r) primers ITS1f/ITS2r39 for the amplification of the ITS-1 region and with the universal primers ITS3f/ITS4r39 for the ITS-2 region was performed [34]. The primers used were extended with the Illumina sequences needed for PCR-based library preparation. The PCR mix (25 µL final volume) contained 2.5 µL of high fidelity PCR buffer (10X), 0.5 µL of a mix of 0.2 mM of each dNTP (ThermoFisher Scientific, Gent, Belgium), 0.5 µM of each primer, 0.1 µL of Platinum^®^ Taq DNA Polymerase high fidelity enzyme (5 U/µL) (ThermoFisher Scientific, Life Technology, Gent, Belgium), and 15.90 µL of Gibco^®^ DNase, RNase, Protease free pure water (Life Technologies, Gent, Belgium). At the end, 5 µL of gDNA (10 ng) were added. Each run was performed with following PCR protocol, i.e., one cycle at 94 °C for 3 min, 35 cycles of 30 sec at 94 °C, 30 sec at 55 °C and 1 min at 72 °C, and at last, 10 min at 72 °C.

After the PCR, all samples were purified with the AMpure^®^ XP PCR purification kit (Agencourt Bioscience corporation, Beverly, MA, USA). The quality and amount of PCR fragments for massive parallel sequencing was verified on a Bioanalyzer 2100 (Agilent Technologies, Amstelveen, the Netherlands). ITS-1 and ITS-2 amplicons from a same sample were mixed together. Equimolar mixes were made according to the size of the peaks observed during the Bioanalyzer analysis. Sequencing was performed by BaseClear (Venlo, The Netherlands) with an Illumina MiSeq, yielding 2 × 300 bp paired-end reads.

### 2.6. Bioinformatics Analysis

The FASTQ sequence files were generated using the Illumina Casava pipeline version 1.8.3 (BaseClear, Venlo, the Netherlands). The first quality assessment was based on the Illumina Chastity filtering. Reads with PhiX control signal and/or short reads below 20 bp (after adapter clipping) were removed using an in-house filtering protocol from BaseClear. Then, a second quality assessment control was done using the tool PRINSEQ-lite quality control v.0.20.4. Finally, an average quality score per sample was obtained with FastQC v.010.1.40 [35]. The sequences which did not pass this quality control were removed for the remainder of the analysis.

To determine the presence of *E. jeanselmei* and other fungal species in indoor air and water samples, sequencing reads were analyzed with an operational taxonomic unit (OTU) classification approach with the Microbial Genomics Module from the CLC Genomic Workbench V8 software (Qiagen Benelux, B.V., KJ Venlo, the Netherlands). Briefly, in order to have comparable read lengths and to remove reads with low coverage, the paired-end reads were firstly treated with three different tools, i.e., “adapter trimming”, “fixed length trimming”, and “filter samples based on the number of reads”. Thus, at the end of the workflow, the OTU clustering was performed only on reads with the same length and a low level of bias.

The OTUs clustering was made with the OTUs clustering tool. The database used as reference was the fungal rDNA sequences Database UNITE V7.1. [36]. Finally, all the OTUs were aligned with the MUSCLE tool and a Neighbor Joining tree was constructed according to the Jukes Cantor nucleotides model.

## 3. Results

### 3.1. Classical Analysis by Culturing

The sampling of the offices and water reservoirs of the air-conditioning systems was performed as elaborated in Materials and Methods. The water and indoor air samples were both incubated on plate in order to detect *E. jeanselmei*, as outlined in the classical monitoring procedures.

For the five water samples collected from the pulse group (PG) of the air-conditioning systems, *E. jeanselmei* was detected in four of them, as seen in Table 1. Expressed in colony forming units per mL (CFU/mL), the range of contamination of the water samples was determined to be between 10 and 100 CFU/mL, as seen in Table 1. *E. jeanselmei* colonies were not observed for the sample collected from the PG 1, as seen in Table 1. In addition, three other determined taxa and three nonsporulating sp. or undetermined species were detected on plates, i.e., *Aspergillus fumigatus*, *Aspergillus puulaeunsis*, and *Penicillium chrysogenum*, as seen in Table 2. As it was done for the water samples, the indoor air samples collected in each office were incubated according to the protocol developed for the detection of *E. jeanselmei* [36,37]. As shown in Table 1, after 21 days of incubation, no *E. jeanselmei* was detected on plate for any of the offices in contact with the air-conditioning system. However, four other determined taxa and four nonsporulating sp. or undetermined species were observed on plate, i.e., *A. alternata*, *Aspergillus* sp., *A. versicolor*, and *P. chrysogenum*, as seen in Table 3.

### 3.2. RT-PCR Detection

The RT-PCR screening was performed on DNA extracted from each air and water sample with the primers specific for the detection of *E. jeanselmei* only [31].

In each run, the PC was amplified with an average quantification cycle (Cq) value of 19.48 ± 1.08 and a melting temperature (Tm) average at 79.58 ± 0.27 °C, corresponding to the expected values previously reported by Libert and colleagues [31]. Four of the five water samples gave a positive signal for *E. jeanselmei* with a Tm average at 79.63 ± 0.30 °C, as seen in Table 1. No signal was observed for the water sample collected from PG1.

Of the 15 indoor air samples tested, four of them gave a positive signal with the expected Tm, i.e., offices 1, 2, and 3 from PG4 and office 1 from PG5, as seen in Table 1. All the NTC were negative. Although the specificity of the *E. jeanselmei* RT-PCR primers has previously been demonstrated [31], the identity of each amplicon (i.e., amplicons from air and water samples) was confirmed as *E. jeanselmei* with a BLASTn analysis of the obtained Sanger sequences. The identify scores obtained with BLASTn for the eight positive samples ranged between 97% for air sample PG4-2 and 100% for the water samples from PGs 2 and 4.

### 3.3. Massive Parallel Sequencing

In order to confirm the results observed in RT-PCR and to obtain a broader view on the uncultivable and/or out-competed fraction in the air and water samples, a massive sequencing analysis was performed on all the DNA samples.

As shown in Table 4, the massive parallel analysis performed on all samples (water and air) generated between 6352 (sample PG1-3) and 29,666 reads (sample PG1-1). Among these reads, between 6797 and 11,602 reads from water samples were clustered into OTUs present in the UNITE database, corresponding to 60.97% (sample PG3) and 99.95% (PG5) of the total reads.

**Figure 1:**
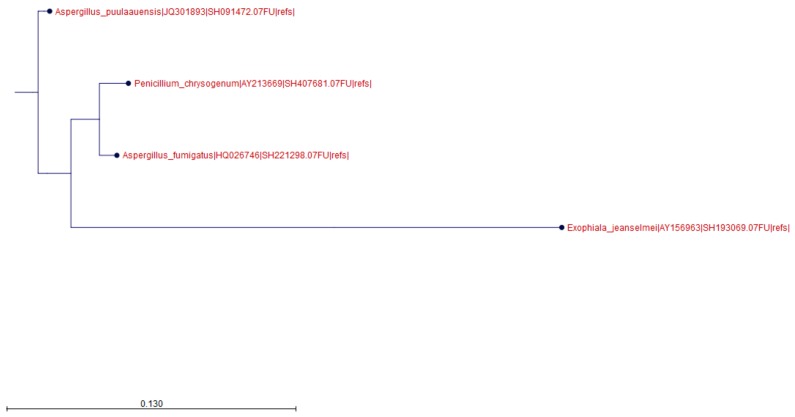
Neighbor Joining tree obtained for the water samples. The Neighbor Joining tree was constructed according to a Jukes Cantor model for the reads obtained for all water samples grouped together.

For PG 2, 3, 4, and 5 (water samples), between 2727 and 5700 reads were grouped into the OTU and identified as *E. jeanselmei* strain AY156963, as seen in Table 4 and Figure 1, the only representative sequence for *E. jeanselmei* included in the UNITE database. The percentage of reads identified as *E. jeanselmei* was between 28% (sample PG2) and 53.69% (sample PG4) of the total number of clustered reads. No reads from PG1 were clustered into the *E. jeanselmei* OTU, as seen in Table 4.

**Figure 2:**
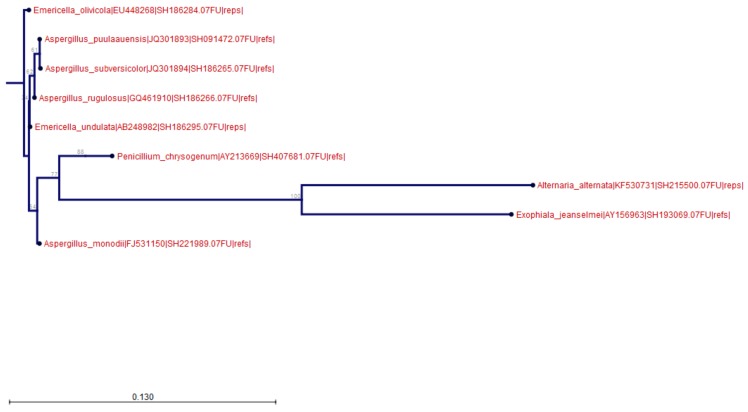
Neighbor Joining tree obtained for the air samples. The Neighbor Joining tree was constructed according to a Jukes Cantor model for the reads obtained for all air samples grouped together.

The number of reads from air samples clustering into OTUs was between 6305 (sample PG1-3) and 25,260 (sample PG2-3). The percentage of reads clustered into OTUs was recorded between 42.95% (sample PG5-2) and 99.99% (PG4-2). In four out of the 15 air samples, *E. jeanselmei* reads were found. Indeed, among the reads clustered into OTUs, between 1248 (sample PG5-1) and 7888 (sample PG4-3) reads were clustered into the *E. jeanselmei* AY156963 OTU, as seen in Table 4 and Figure 2. Expressed in %, between 10.99% (sample PG5-1) and 60.74% (sample PG4-1) of reads grouped into OTUs were identified as *E. jeanselmei* OTU. These samples were collected in four offices connected to two PGs i.e., offices 1, 2, and 3 of PG4 and office 1 of PG5, as seen in Table 4. For the samples of PG1, 2, and 3, no reads were correctly clustered into the *E. jeanselmei* OTU, as seen in Table 4.

In addition to *E. jeanselmei*, as shown in Table 2 and Table 3, additional OTUs were detected besides *E. jeanselmei*, i.e., three in water samples, *A. fumigatus*, *A. puulaaeunsis* and *P. chrysogenum*, as seen in Figure 1, and nine in air samples, i.e., *A. alternata*, *Aspergillus monodii*, *A. puulaaeunsis*, *Aspergillus rugulosus*, *Aspergillus subversicolor*, *Aspergillus* sp., *Emericella olivicola*, *Emericella undulata*, and *P. chrysogenum*, as seen in Figure 2.

## 4. Discussion

*E. jeanselmei* is commonly found in moist environments, especially in water reservoirs of air-conditioning systems [37,38,39], but it has not yet been detected in indoor air of offices in contact with these air-conditioning systems. This could be explained by the classical use of culture-based protocols for the monitoring and investigation of fungal indoor air contamination. In this study, it was investigated whether the presence of *E. jeanselmei* in indoor air of these offices could be demonstrated using molecular methods, thereby avoiding the bias created by competition between species, incubation conditions, and the presence of uncultivable taxa when using culture-based methods.

Herein, a sampling was organized in different offices in contact with an air-conditioning system for which contamination by *E. jeanselmei* was previously detected. First, water samples from the water-reservoir from five PGs were collected in order to verify the presence of *E. jeanselmei* inside the system, i.e., in the water samples. These samples were analyzed using the classical protocol based on culture and microscopic identification [32], and molecular methods including RT-PCR [31] and massive parallel sequencing analysis. All samplings were performed between February and March, i.e., at the yearly restart of the air-conditioning systems, in the framework of the air-conditioning water monitoring. This monitoring is carried out yearly before the water decontamination (part of the maintenance of the system), except for PG1, which had been maintained (including decontamination and draining) already before the time of sampling. The classical analysis of the water samples showed that all of the investigated water systems were contaminated with *E. jeanselmei*, except the one from PG1, as seen in Table 1. The maintenance performed on PG1 explained the absence of *E. jeanselmei*. The results of the RT-PCR as well as of the massive parallel sequencing confirmed these positive detections of *E. jeanselmei* in water samples, as seen in Table 1 and Table 4, of PG2, 3, 4, and 5. Consistently, neither a signal nor a read was observed for the PG1 sample with RT-PCR or massive parallel sequencing, respectively.

In parallel, indoor air samples were collected in 15 offices in contact with the air-conditioning systems. For each system, three offices were selected based on their distance to the pulse group, i.e., one close to the pulse group, one far from the pulse group and one in the middle of the two others. This was done in order to define whether a distance effect could impact the detection of *E. jeanselmei* in air. As performed for the water samples, each air sample was analyzed with classical and molecular approaches. No *E. jeanselmei* colonies were observed for the air samples incubated on plates, even after 21 days of incubation. However, positive signals for *E. jeanselmei* were observed by RT-PCR in air samples from the offices 1, 2, and 3 linked to PG4, and in office 1 of PG5. These positive detections were obtained for the air-conditioning systems where the highest water contamination was observed on plate (100 CFU/mL for PG4 and 15 CFU for PG5, compared to 10 for PG2 and PG3). In PG5, *E. jeanselmei* was observed only in the office the closest located to the PG, not in office 2 and 3 of PG5. Moreover, according to the RT-PCR results, a gradient of distance from the closest to the most distantly located offices was observed for the PG4 with Cq values ranging between 23.79 ± 0.92 for PG4-1 and 34.06 ± 1.12 for PG4-3. The distance between the source of the contamination and the sampling site and the contamination load could probably affect the spreading of biological material originating from *E. jeanselmei* as was observed for other species from indoor and outdoor environment [40]. It would be interesting to study the size and the form of the airborne biological compounds of *E. jeanselmei* involved to determine their aerosolizing distance in indoor air to verify this hypothesis.

The results of the massive parallel sequencing confirmed the ones obtained by RT-PCR. The obtained reads were clustered onto the UNITE database as reference, which contained 6825 reference ITS sequences at the date of research. Reads were clustered onto the *E. jeanselmei* OTU for samples PG4-1, PG4-2, PG4-3, and PG5-1. For these samples, positive RT-PCR signals were obtained. The percent of reads clustering into *E. jeanselmei* decreased with increasing distance between the sampling site and the PG location. This is a similar trend as was observed with the Cq values in the RT-PCR analysis. This points towards a semiquantitative nature of the massive parallel sequencing analysis.

Massive parallel sequencing was performed to obtain a broader view on the uncultivable species present in the water and air samples. Indeed, besides *E. jeanselmei* in indoor air and water samples, *A. alternata*, *A. fumigatus*, *A. puulaaeunsis*, *A. versicolor*, *P. chrysogenum*, as well as *Aspergillus* sp., an undetermined sp. and a nonsporulating sp., were detected on plate with the classical culture-based protocol. These taxa are commonly observed in indoor environment [1,33]. In the water samples, the sequencing and classical approaches yielded the same species. However, besides *E. jeanselmei*, five additional OTUs were retrieved from air with the massive parallel sequencing which were not detected by the classical methods in the air samples, i.e., *A. monodii*, *A. puulaaeunsis*, *A. rugulosus*, *E. olivicola*, and *E. undulata*. This means that of the species retrieved in the OTU analysis from air samples, only two were confirmed by the culturing approach, i.e., *A. alternata* and *P. chrysogenum*. *A. versicolor* was found in the air samples with the classical methods, but not with the massive parallel sequencing. However, two species from the same phylogenetic species, i.e., Versicolores were observed in the OTUs, i.e., *A. puulaaeunsis* and *A. subversicolor*. It should be noted that poor read quality could affect the correct determination of closely related species, e.g., for the species from the Versicolores complex and especially *A. versicolor* and *A. puulaauensis*, for which the ITS region differs only in a few nucleotides.

During the clustering, between 0.01% and 57.05% of reads were not included in the OTUs. This can be linked either to reads that did not comply with the quality criteria or to reads that correspond to species that are not covered in the UNITE database or chimeric reads. This indicates that the massive parallel sequencing approach would benefit from improved and extended ITS databases in the future. The lower number of clustered reads obtained for some samples, especially for samples PG3 and PG5-2, could have an impact on the results obtained during the diversity analysis. However, based on the RT-PCR results, these potential biases have not affected the detection of *E. jeanselmei*.

The literature available on *E. jeanselmei* detected in air is very scarce. In 2017, Kirtsideli and coworkers [20] detected this fungus for the first and only time in air, both outdoors (coastal areas, landscape, streets) and indoors (public building, empty houses). They showed that there was a higher concentration outdoors than indoors, when analyzing sampling data of 2012–2015 for the arctic settlement Tiksi, which suggests that the fungus could have a major outdoor source. During our monitoring campaign, unfortunately, no outdoor samples were taken. However, it has to be questioned whether the outdoor sampling on one day would be representative to explain a possible outdoor source of *E. jeanselmei* contamination, as was described for the 2012–2015 period in the arctic settlement [20]. Moreover, to the best of our knowledge, there are currently no reports available describing the detection using classical methods of *E. jeanselmei* in outdoor air samples in Belgium. Additionally, given the fact that *E. jeanselmei* was only found in four of the 15 sampled offices, with a clear correlation with a high PG contamination, we tend to believe that the source of air contamination in the sampled offices is the PG of the air-conditioning system. In 2010, Huang and his team reported a case of hypersensitivity pneumonitis caused by *E. jeanselmei* in a person who was a regular visitor of a specific sauna [41]. According to their conclusion, the way of contamination was probably by the sauna steam [41], due to the presence of the black yeast in the water of the sauna visited by the patient. However, as this black yeast can be present in dry indoor air from buildings, this could also be a possible way of infection.

The detection of *E. jeanselmei* with molecular tools, but not with the classical method, confirms the hypothesis that *E. jeanselmei* could be present in the suppressed or uncultivable fraction of the indoor airborne fungal community. This indicates that airborne infection of *E. jeanselmei* is possible. As shown in this study, RT-PCR and massive parallel sequencing analysis can improve the monitoring of indoor air, especially in the field of fungal contamination. Indeed, the use of universal primers amplifying the ITS region of a large panel of fungal species increases the number of species that can be simultaneously detected during a monitoring. This is limited to one or a few a priori selected species with RT-PCR [42]. Another advantage of massive parallel sequencing is that the detection and identification are performed simultaneously, while the confirmation of RT-PCR results requires sequencing of the amplicon if the tool is not fully specific. However, massive parallel sequencing is more time-consuming and requires bioinformatic expertise for the data interpretation, which is not the case for RT-PCR. In addition to the use of molecular tools, the detection of *E. jeanselmei* was probably also facilitated by the use of this particular air collector, which is different from the one generally used for culture-dependent monitoring. The Coriolis^®^ µ allows collecting a high volume of air (1.5 m^3^) directly into collection liquid, which can be incubated on a plate or analyzed with molecular tools without a culturing step, as was done in this study.

## 5. Conclusions

The results obtained in this study, taking *E. jeanselmei* as a case study, demonstrate that molecular methods, such as RT-PCR or massive parallel sequencing, combined with an appropriate air sampler, can increase the data retrieved on the fungal community diversity in indoor air, compared to the classical culture-based methods. More investigations on indoor air should be performed in order to improve the knowledge on indoor airborne fungi contamination, particularly on the suppressed and/or uncultivable fraction. Eventually, an improved insight on this diversity will contribute to the understanding of public health problems such as respiratory diseases (asthma, rhinitis, or other chronicle respiratory infections) and sick buildings syndrome observed in many indoor environments, and to taking the appropriate preventive measures.

## Figures and Tables

**Table 1:** Detection of *E. jeanselmei* in indoor air and water in air-conditioning systems: Classical analysis and RT-PCR.

Sample ID *	Pulse Group	Office	Sample Type	Analysis Method		
				Culture	RT-PCR	
				CFU/mL ^1^	Tm (°C) ^2^	Cq ^3^
PG1 ^4^	1		Water ^5^	0	N/A	N/A
PG1-1	1	1	Air	0	N/A	N/A
PG1-2	1	2	Air	0	N/A	N/A
PG1-3	1	3	Air	0	N/A	N/A
PG2	2		Water ^5^	10	79.53 ± 0.12	28.00 ± 1.35
PG2-1	2	1	Air	0	N/A	N/A
PG2-2	2	2	Air	0	N/A	N/A
PG2-3	2	3	Air	0	N/A	N/A
PG3	3		Water ^5^	10	79.79 ± 0.32	28.39 ± 0.93
PG3-1	3	1	Air	0	N/A	N/A
PG3-2	3	2	Air	0	N/A	N/A
PG3-3	3	3	Air	0	N/A	N/A
PG4	4		Water ^5^	100	79.93 ± 0.05	20.32 ± 1.98
PG4-1	4	1	Air	0	79.44 ± 0.10	23.79 ± 0.92
PG4-2	4	2	Air	0	79.49 ± 0.03	24.49 ± 0.23
PG4-3	4	3	Air	0	79.38 ± 0.03	34.05 ± 1.75
PG5	4		Water ^5^	15	79.34 ± 0.23	27.36 ± 1.12
PG5-1	5	1	Air	0	79.39 ± 0.02	27.62 ± 0.63
PG5-2	5	2	Air	0	N/A	N/A
PG5-3	5	3	Air	0	N/A	N/A

* PG = pulse group, i.e., the part of the air-conditioning system containing the machinery to pulse the air and the water-reservoir needed to maintain the required hygrometry level; the first number refers to the number of the PG (five in total); the number after the ‘-’ refers to the office connected to the PG, at different distances (see Materials and Methods). ^1^ CFU/mL defined as the number of colonies of *E. jeanselmei* on plate after 21 days of incubation at 37 °C. ^2^ Tm defined as the average of the melting temperature (°C) observed for each RT-PCR amplicon obtained during the qPCR SYBR^®^Green analysis performed in duplicate in two independent runs. ^3^ Cq defined as the average quantification cycle observed for each RT-PCR amplicon obtained during the qPCR SYBR^®^Green analysis performed in duplicate in two independent runs. ^4^ PG1 was maintained before the sampling. ^5^ One sample per pulse group was analyzed.

**Table 2:** Fungal contamination in water samples, other than *E. jeanselmei*: Classical analysis and sequencing analysis comparison.

Sample *	Classical Analysis ^1^		NGS	
	Species	CFU/mL	Species	Abundance of Reads Per OTU ^2^ (%)
PG1	*Penicillium chrysogenum*	5	*Penicillium chrysogenum*	100
PG2	*Penicillium chrysogenum*	5	*Penicillium chrysogenum*	60.64
	*Aspergillus puulaaeunsis*	1	*Aspergilluspuulaaeunsis*	11.27
	Nonsporulating sp.	1		
	*Aspergillus fumigatus*	4	*Aspergillus fumigatus*	36.34
PG3	*Penicillium* *chrysogenum*	5	*Penicillium* *chrysogenum*	30.15
	Nonsporulating sp.	2		
	Undetermined species	1		
PG4	*Aspergillus fumigatus*	2	*Aspergillus fumigatus*	12.19
PG5	*Penicillium chrysogenum*	3	*Penicillium chrysogenum*	23.30
			*Aspergillus puulaaeunsis*	11.86

* PG = pulse group, i.e., the part of the air-conditioning system containing the machinery to pulse the air and the water-reservoir needed to maintain the required hygrometry level; the first number refers to the number of the PG (5 in total). ^1^ CFU/mL defined as the number of colonies observed on plate after five days and 21 days of incubation at 37 °C, *E. jeanselmei* excluded. The data for *E. jeanselmei* is presented in Table 1. ^2^ The massive parallel sequencing data were analyzed with the CLC Genomic Workbench software (Qiagen Benelux, B.V., KJ Venlo, the Netherlands) and the Microbial Genomics Module. The database used as reference was the UNITE database^36.^

**Table 3:** Fungal contamination in air samples, other than *E. jeanselmei*: Classical analysis and sequencing analysis comparison.

Samples *	Classical Analysis ^1^		NGS ^2^	
	Species	CFU/mL	Species	Abundance of Reads Per OTU ^2^ (%)
PG1-1	*Alternaria alternata*	5	*Alternaria alternata*	21.86
	*Aspergillusversicolor*	3	*Aspergillus subversicolor*	11.49
	*Penicillium chrysogenum*	9	*Penicillium chrysogenum*	66.65
PG1-2	*Alternaria alternata*	3	*Alternaria alternata*	24.72
	*Aspergillus* sp.	2	*Aspergillus monodii*	7.70
	Nonsporulating sp.	1	*Aspergillus rugulosus*	4.43
	*Penicillium chrysogenum*	7	*Penicillium chrysogenum*	62.40
PG1-3	*Alternaria alternata*	2	*Alternaria alternata*	31.28
	Undetermined species	1	*Aspergillus monodii*	15.93
	Nonsporulating sp.	1	*Aspergillus rugulosus*	12.47
	*Penicillium chrysogenum*	7	*Penicillium chrysogenum*	39.58
PG2-1	Undetermined sp.	2	*Emericella olivicola*	16.22
	*Penicillium chrysogenum*	6	*Penicillium chrysogenum*	71.44
			*Emericella undulata*	12.35
PG2-2	*Alternaria alternata*	2	*Alternaria alternata*	24.59
	*Penicillium chrysogenum*	6	*Penicillium chrysogenum*	75.41
PG2-3	*Alternaria alternata*	4	*Alternaria alternata*	32.28
	*Aspergillus versicolor*	5	*Aspergillus subversicolor*	32.09
	*Aspergillus* sp.	1	*Aspergillus monodii*	24.26
	Nonsporulating sp.	1	*Aspergillus puulaaeunsis*	11.37
PG3-1	*Alternaria alternata*	4	*Alternaria alternata*	53.50
	*Penicillium chrysogenum*	5	*Penicillium chrysogenum*	46.50
PG3-2	*Alternaria alternata*	5	*Alternaria alternata*	71.96
	Undetermined sp.	2	*Emericella olivicola*	16.41
			*Emericella undulata*	11.63
PG3-3	*Aspergillus* sp.	2	*Aspergillus monodii*	11.18
			*Aspergillus rugulosus*	19.56
			*Aspergillus subversicolor*	20.40
	*Penicillium chrysogenum*	6	*Penicillium chrysogenum*	48.85
PG4-1	*Alternaria alternata*	1	*Alternaria alternata*	26.18
	*Aspergillus versicolor*	1	Undertermined sp.	13.07
	Nonsporulating sp.	1		
PG4-2	*Alternaria alternata*	3	*Alternaria alternata*	17.48
	*Penicillium chrysogenum*	9	*Penicillium chrysogenum*	40.88
	Undetermined sp.			
PG4-3	*Aspergillus versicolor*	4	*Aspergillus subversicolor*	29.12
	Nonsporulating sp.	1	*Aspergillus puulaaeunsis*	24.38
			*Aspergillus monodii*	7.17
PG5-1	*Penicillium chrysogenum*	7	*Penicillium chrysogenum*	64.57
	Undetermined sp.	1	*Emericella undulata*	13.98
	Nonsporulating sp.	1	*Emericella olivicola*	10.46
PG5-2	*Alternaria alternata*	4	*Alternaria alternata*	31.33
	*Aspergillus versicolor*	4	*Aspergillus subversicolor*	32.55
			*Aspergillus puulaaeunsis*	36.12
	Nonsporulating sp.	2		
PG5-3	*Aspergillus versicolor*	6	*Aspergillus subversicolor*	39.24
	*Penicillium chrysogenum*	8	*Penicillium chrysogenum*	60.76

* PG = pulse group, i.e., the part of the air-conditioning system containing the machinery to pulse the air and the water-reservoir needed to maintain the required hygrometry level; the first number refers to the number of the PG (5 in total); the number after the ‘-‘ refers to the office connected to the PG, at different distances (see Materials and Methods). ^1^ CFU/mL defined as the number of colonies observed on plate after five days and 21 days of incubation at 37 °C. ^2^ The massive parallel sequencing data were analyzed with the CLC Genomic Workbench software (Qiagen Benelux, B.V., KJ Venlo, the Netherlands) and the Microbial Genomics Module. The database used as reference was the UNITE database^36.^

**Table 4:** Detection of *E. jeanselmei* in indoor air and water in air-conditioning systems: sequencing data and clustering results.

	Pulse Group	Office ^1^	Sample Type	Total Number of Reads ^2^	Total of Reads Clustered ^3^	% Reads Clustered ^4^	Abundance of Reads into *E. jeanselmei* OTU ^5^	% Reads into *E. jeanselmei* OTU (%) ^6^
PG1 ^7^	1		Water ^8^	13,124	11,602	88.40	0	0
PG1-1	1	1	Air	29,666	23,545	79.37	0	0
PG1-2	1	2	Air	19,753	19,603	99.24	0	0
PG1-3	1	3	Air	6352	6305	99.26	0	0
PG2	2		Water ^8^	9755	9741	99.86	2727	28.00
PG2-1	2	1	Air	28,487	20,928	73.47	0	0
PG2-2	2	2	Air	15,193	15,093	99.34	0	0
PG2-3	2	3	Air	28,338	25,260	89.14	0	0
PG3	3		Water ^8^	11,148	6797	60.97	2278	33.51
PG3-1	3	1	Air	9154	9003	98.35	0	0
PG3-2	3	2	Air	9474	9434	99.58	0	0
PG3-3	3	3	Air	9474	9165	96.74	0	0
PG4	4		Water ^8^	10,675	10,616	99.45	5700	53.69
PG4-1	4	1	Air	8402	8360	99.50	5078	60.74
PG4-2	4	2	Air	16,838	16,837	99.99	7011	41.64
PG4-3	4	3	Air	20,286	20,059	98.88	7888	39.32
PG5	4		Water ^8^	9302	8458	90.93	4453	52.65
PG5-1	5	1	Air	12,288	11,355	92.41	1248	10.99
PG5-2	5	2	Air	16,337	7016	42.95	0	0
PG5-3	5	3	Air	9328	9320	99.91	0	0

The massive parallel sequencing data were analyzed with the CLC Genomic Workbench software (Qiagen Benelux, B.V., KJ Venlo, the Netherlands) and the Microbial Genomics Module. The database used as reference was the UNITE database^36^. ^1^ Three offices per pulse group (PG) were sampled according to their distance to the PG. Office 1 corresponds to the closest located office to the PG, office 3 the most distantly located office to the PG, and office 2 correspond to the office located between the two others. ^2^ The total number of reads observed for each sample. ^3^ Number of total reads grouped in the 10 OTUs detected, i.e., *A. alternata*, *Aspergillus fumigatus, Aspergillus monodii*, *Aspergillus puulaaeunsis*, *Aspergillus rugulosus*, *Aspergillus subversicolor*, *Emericella olivicola*, *Emericella undulata*, *P. chrysogenum*, and *E. jeanselmei*. ^4^ Percentage obtained as the ratio between the number of reads grouped into the 10 OTUs and the total number of reads observed for each sample. ^5^ Number of reads corresponding to OTU identified as *E. jeanselmei*. ^6^ Percentage obtained as the ratio between the number of reads corresponding to the *E. jeanselmei* OTU and the total number of clustered reads. ^7^ PG1 maintained before the sampling. ^8^ One sample per pulse group was analyzed.
